# Structural and biochemical characterization of the class II fructose-1,6-bisphosphatase from *Francisella tularensis*

**DOI:** 10.1107/S2053230X20013370

**Published:** 2020-10-23

**Authors:** Anna I. Selezneva, Hiten J. Gutka, Nina M. Wolf, Fnu Qurratulain, Farahnaz Movahedzadeh, Celerino Abad-Zapatero

**Affiliations:** a Institute for Tuberculosis Research, University of Illinois at Chicago, Chicago, Illinois, USA; b Department of Pharmaceutical Sciences, University of Illinois at Chicago, Chicago, Illinois, USA; c Center for Biomolecular Sciences, University of Illinois at Chicago, Chicago, Illinois, USA

**Keywords:** class II FBPases, fructose-1,6-bisphophatase, *Mycobacterium tuberculosis*, *Francisella tularensis*, antibiotic targets

## Abstract

The crystal structure of the class II fructose-1,6-bisphosphatase (FBPaseII) from the important pathogen *Francisella tularensis* is presented at 2.4 Å resolution. Its structural and functional relationships to the closely related phosphatases from *Mycobacterium tuberculosis* (*Mt*FBPaseII) and *Escherichia coli* (*Ec*FBPaseII) and to the dual phosphatase from *Synechocystis* strain 6803 are discussed. FBPaseII from *F. tularensis* (*Ft*FBPaseII) was crystallized in a monoclinic crystal form (space group *P*2_1_, unit-cell parameters *a* = 76.30, *b* = 100.17, *c* = 92.02 Å, β = 90.003°) with four chains in the asymmetric unit. Chain *A* had two coordinated Mg^2+^ ions in its active center, which is distinct from previous findings, and is presumably deactivated by their presence. The structure revealed an approximate 222 (*D*_2_) symmetry homotetramer analogous to that previously described for *Mt*FBPaseII, which is formed by a crystallographic dyad and which differs from the exact tetramer found in *Ec*FBPaseII at a 222 symmetry site in the crystal. Instead, the approximate homotetramer is very similar to that found in the dual phosphatase from *Synechocystis*, even though no allosteric effector was found in *Ft*FBPase. The amino-acid sequence and folding of the active site of *Ft*FBPaseII result in structural characteristics that are more similar to those of the previously published *Ec*FBPaseII than to those of *Mt*FBPaseII. The kinetic parameters of native *Ft*FBPaseII were found to be in agreement with published studies. Kinetic analyses of the Thr89Ser and Thr89Ala mutations in the active site of the enzyme are consistent with the previously proposed mechanism for other class II bisphosphatases. The Thr89Ala variant enzyme was inactive but the Thr89Ser variant was partially active, with an approximately fourfold lower *K*_m_ and *V*_max_ than the native enzyme. The structural and functional insights derived from the structure of *Ft*FBPaseII will provide valuable information for the design of specific inhibitors.

## Abstract 2

The structure of the class II fructose-1,6-bisphosphatase from *F. tularensis* is presented together with kinetic data for the wild type and the active-site mutant Thr89Ser. The structure reveals a tetramer of four identical subunits with approximate 222 symmetry. The structure is related to the homologous structure from the related pathogen *Mycobacterium tuberculosis*.

## 1. Introduction   

*Francisella tularensis*, which causes tularemia, is one of the most virulent and deadly pathogens to be registered as a biological weapon (Dennis *et al.*, 2001 ▸). Highly pathogenic biovars of *F. tularensis* have been discovered in all parts of the world. The first isolation of *F. tularensis* subsp. *tularensis* occurred in Europe (Guryčová, 1998 ▸). The pathogen is capable of infecting various types of cells from more than 250 hosts, but in humans it is thought to replicate intracellularly to high densities mainly in macrophages (Santic *et al.*, 2006 ▸; Sjöstedt, 2006 ▸; Keim *et al.*, 2007 ▸). Clinical outcomes in the treatment of tularemia often correlate poorly with the results from *in vitro* experiments on susceptibility to antibiotics.

Traditionally, antibiotic therapies for tularemia have been determined empirically by testing the bactericidal or bacteriostatic effect using whole cells, and most of the useful drugs were found to be toxic or only resulted in a bacteriostatic effect. Therefore, more effective and less toxic drugs are in demand. One approach to pursue a bactericidal effect is the rational design of drugs that target the essential metabolic pathways. It has been demonstrated that gluconeogenesis, but not glycolysis, is essential for the intracellular growth and virulence of *F. tularensis* (Brissac *et al.*, 2015 ▸). Genetic screens resulted in the identification of the gluconeogenic enzyme class II fructose-1,6-bisphosphatase (*Ft*FBPaseII), the product of the *glpX* gene, as one of the most important contributors to the virulence of *F. tularensis* strains SchuS4 and LVS (Kadzhaev *et al.*, 2009 ▸). Further work has demonstrated that *Francisella* relies on the supply of amino acids from the host as major gluconeogenic substrates for intracellular growth (Ziveri *et al.*, 2017 ▸), highlighting the importance of the *Ft*FBPaseII enzyme; no other FBPases have been characterized in *F. tularensis*. Thus, targeted inactivation of *Ft*FBPaseII should prevent the bacterium from producing fructose 6-phosphate (F6P) during intracellular growth, regardless of the specific gluconeogenic host-derived carbon source that is available to the pathogen (Radlinski *et al.*, 2018 ▸). F6P is a precursor of the pentose phosphate pathway and is necessary for the *de novo* synthesis of molecules that are essential for the survival of *F. tularensis* in the host, including lipopolysaccharides, peptidoglycan and pentose phosphates.

FBPases are a broad group of enzymes consisting of five different classes (I–V) which share similarities in amino-acid sequence and consequently in structural fold. Prokaryotic FBPases are diverse and contain members of all five classes, while eukaryotic FBPases are mostly limited to class I, which provides the opportunity for the design of bacterial FBPase inhibitors that are harmless to mammalian enzymes. Similarity in the structure of all classes of FBPases is displayed by the presence of two adjacent α/β domains, one of which is usually two-layered (α–β ; CATH code 3.30.540), with the other being three-layered (α–β–α ; CATH code 3.40.190). Together, these domains form a five-layered sandwich-like structure (α–β–α–β–α), in which the β-sheets are positioned nearly orthogonal to each other. Class-specific structural differences include the number of strands in each sheet, the boundaries of the strands and the topology connecting them. A number of FBPase class II structures have been reported to date of both apo enzymes and complexes with different effectors. *Ft*FBPaseII has high (∼50%) amino-acid sequence identity and is assumed to be structurally homologous to other bacterial class II FBPases, such as those from *Mycobacterium tuberculosis* (*Mt*FBPaseII) and *Escherichia coli* (*Ec*FBPaseII) and in particular the dual enzyme fructose-1,6-/sedoheptulose-1,7-bisphophatase from the cyanobacterium *Synechocystis* strain 6803. Structures of these three enzymes in the apo form and bound to various ligands are available from the Protein Data Bank: PDB entries 6ayy, 6ayu and 6ayv for *Mt*FBPaseII (Wolf *et al.*, 2018 ▸), PDB entries 3big, 3bih and 3d1r for *Ec*FBPaseII (Brown *et al.*, 2009 ▸), and PDB entries 3roj and 3rpl for the *Synechocystis* dual enzyme (Feng *et al.*, 2014 ▸).

Despite the increasing number of available structures, there are still biochemical and functional questions that need to be addressed in order to successfully establish structure-guided drug-design projects targeting *Ft*FBPaseII. Some of the ambiguities relate to the native oligomeric state of the enzyme, the enzyme stability in solution and the exact nature of the metal cofactors and their stoichiometry in the active site.

The functional quaternary structures of class II FBPases in solution are diverse, ranging from a proposed dimer for *Ec*FBPaseII (Brown *et al.*, 2009 ▸) to a described tetramer for *Mt*FBPaseII (Wolf *et al.*, 2018 ▸). These enzymes were found as tetrameric aggregates in the crystal, with monomers and dimers in the asymmetric unit, respectively. To date, only the crystal structure of the *Synechocystis* dual enzyme in the presence of the allosteric effector AMP has been found to contain a full tetramer in the asymmetric unit (PDB entry 3rpl; Feng *et al.*, 2014 ▸). *Ft*FBPaseII has been observed as a mixture of dimers and tetramers in solution, with the larger tetrameric state being found at higher protein concentrations (Gutka *et al.*, 2017 ▸). The structure-based design of compounds aimed at disruption of the native oligomeric state of *Ft*FBPaseII could be a promising strategy for successful rational drug design in order to bypass the high polarity of compounds binding in the active site.

A preference for Mn^2+^ as a metal cofactor together with weak Li^+^ sensitivity makes *Ft*FBPaseII similar to *Ec*FBPaseII (Gutka *et al.*, 2017 ▸; Brown *et al.*, 2009 ▸). However, the majority of known FBPases can be activated by Mg^2+^ or by a mixture of Mg^2+^ and Mn^2+^, and are readily deactivated by Li^+^. Surprisingly, the presence of Mg^2+^ in the active site of *Ft*FBPaseII completely inhibits activity (Gutka *et al.*, 2017 ▸). The structural basis for these specificities is unknown and could also be exploited for the purpose of designing compounds that are highly specific for *Ft*FBPaseII. Here, we present the first crystal structure of *Ft*FBPaseII and its comparison with other relevant members of the class II bisphosphatases in order to provide insights into the metal dependence, protein oligomeric state and enzymatic activity of *Ft*FBPaseII.

## 2. Materials and methods   

### 2.1. *Ft*FBPaseII protein: mutagenesis, overexpression in *E. coli*, purification and storage   

A previously engineered construct of the *F. tularensis glpX* gene containing an N-terminal His_6_ tag in pET-15b vector was used for overexpression of the *Ft*FBPaseII enzyme in *E. coli* (Gutka *et al.*, 2017 ▸). The T89S and T89A mutants in the *Ft*FBPaseII active site were obtained using the QuikChange II site-directed mutagenesis kit (Agilent Technologies) following the manufacturer’s recommended procedure. Forward primers and their reverse complements were designed to replace the threonine at position 89 of *Ft*FBPaseII with alanine (T89A; 5′-CCCGCTGGAAGGTGCGACCATTACCAGC-3′) and serine (T89S; 5′-CCCGCTGGAAGGTTCGACCATTACCAGC-3′). In the mutant genes, the ACG codon for Thr89 was replaced by GCG for alanine and TCG for serine. Substitutions were verified by DNA sequencing. Overexpression of the wild-type and mutant *Ft*FBPaseIIs and the purification of the proteins from *E. coli* was performed as described previously (Gutka *et al.*, 2017 ▸). All purification procedures were performed at 277 K. The enzyme was stored as single-use aliquots at 193 K until further use. Details are summarized in Supplementary Table S1.

### 2.2. Enzymatic assays   

The protein for enzymatic assays was purified, concentrated to at least 10 mg ml^−1^ and stored in 20 m*M* Tris–HCl pH 8.0, 50 m*M* KCl, 7% glycerol. A previously established enzymatic assay based on two coupled reactions using phosphogluco­isomerase and glucose-6-phosphate dehydrogenase in the presence of NADP^+^ was used to determine the activity of *Ft*FBPaseII (Rittmann *et al.*, 2003 ▸; Gutka *et al.*, 2011 ▸). The ernzymes for the assay were purchased from Sigma–Aldrich. The reactions were carried out in buffer consisting of 50 m*M* KCl, 50 m*M* MnCl_2_, 20 m*M* Tris–HCl pH 8.0 and the substrate fructose 1,6-bisphosphate (F16BP) at various concentrations. MnCl_2_ was added to the reaction directly before the substrate. The assay was performed in triplicate and the activities of wild-type and mutant *Ft*FBPaseII were tested at a final concentration of 50 n*M*. A reaction lacking *Ft*FBPaseII served as a negative control. The activity of *Ft*FBPaseII in the assay was monitored by the formation of NADPH at 340 nm. Data were fitted to a Michaelis–Menten kinetic model using *Prism* to calculate *K*_m_ and its error. The ratio *V*_WT_/*V*_T89S_ for the median values of the reaction speed obtained from kinetic analysis was plotted against the corresponding substrate concentrations and fitted using *Prism* in order to analyze the changes in *K*_m_ introduced by the mutation (Eisenthal *et al.*, 2007 ▸).

### 2.3. Crystallization   

The purified protein (10 mg ml^−1^) in 20 m*M* Tricine pH 7.8, 50 m*M* KCl, 1 m*M* MgCl_2_, 0.1 m*M* DTT, 15% glycerol was combined with F6P at 1 m*M* (a 1:4 protein:ligand ratio). The complex and apo samples were incubated overnight with the ligand at 4°C. An initial crystal (crystal *A*) suitable for data collection was grown in ammonium sulfate, 0.1 *M* bis-Tris pH 5.5, 17% PEG 10 000 at a protein concentration of 10 mg ml^−1^ in the presence of the substrate F16BP at 1 m*M* concentration. Subsequent crystallization trials were carried out at 291 K using the sitting-drop vapor-diffusion method. Drops were prepared by combining the enzyme solution with precipitant in a 1:1 volume ratio and were then equilibrated against 0.1 ml reservoir solution. The PEGs, PACT, pHClear, Classics and JCSG+ Suites (Qiagen) crystallization screens were used as the initial screening conditions. A second crystal (crystal *B*), which diffracted to higher resolution, was obtained using 0.2 *M* sodium formate, 20% PEG 3350 in the presence of F6P. Data are summarized in Supplementary Table S2.

### 2.4. Data collection and processing   

Crystals were harvested from the drops, soaked for 15–30 s in a cryoprotectant solution consisting of reservoir solution plus 20% glycerol, mounted on nylon loops and flash-cooled by immersion in liquid nitrogen. X-ray diffraction data were collected near liquid-nitrogen temperature on the 21-ID-G beamline at the Advanced Photon Source, Argonne National Laboratory, Illinois, USA. Diffraction data sets were collected from several crystals at a wavelength of 0.97856 Å using a MAR300 detector at the Life Sciences Collaborative Access Team (LS-CAT) or Southeastern Regional Collaborative Access Team (SER-CAT) at the Advanced Photon Source (λ = 1.0000 Å).

For crystal *A*, 240 frames were collected (1° scan width and 1 s exposure per frame) at a crystal-to-detector distance of 280 mm, but owing to crystal decay only 200 frames were processed to a nominal resolution of 2.7 Å. A similar data-collection strategy was used for crystal *B*: a 180° wedge including a total of 360 frames was collected with a 0.5° scan width and an exposure time of 4 s per frame at the same crystal-to-detector distance. The data were indexed and scaled with the *HKL*-2000 package (Otwinowski & Minor, 1997 ▸).

### 2.5. Structure solution, refinement and subsequent analysis   

**Figure 1:**
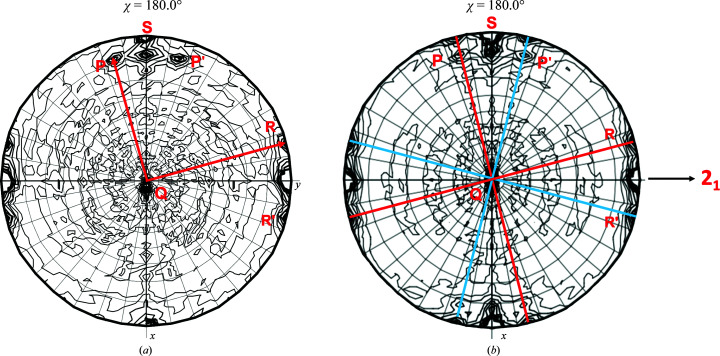
Noncrystallographic symmetry in the crystals of *Ft*FBPase. The self-rotation function (SRF; χ = 180°) of the crystallographic data for the monoclinic crystals of *Ft*FBPaseII was calculated and is shown. (*a*) Crystal *A*. It was impossible to characterize/refine a twinning law or fraction from this crystal. Uncorrected structure factors were used to obtain an initial structure. The SRF indicated the presence of two sets of nearly orthogonal twofold axes (*P*, *Q*, *R* and *P*′, *Q*′, *R*′). (*b*) The diffraction data from crystal *B* were used to refine the structure of *Ft*FBPaseII. The SRF consists of two sets of three nearly orthogonal twofold axes. The lack of perfect centrosymmetric symmetry reflects the two twinning fractions of 0.567 and 0.433 for the crystal. The structure refinement included refinement of the twinning fractions (Table 1 ▸). The two sets of nearly orthogonal twofold peaks of the SRF were shown to correspond to the following dimer relationships by model calculations using the appropriate structure factors as follows: *A*–*B*/*C*–*D* (*P*), *A*–*D*/*B*–*C* (*Q*) and *A*–*C*/*B*–*D* (*R*). In addition to these orientations of the tetramers, there could be other orientations in the crystal along the planes indicated in (*b*), as indicated by the blue and red lines. The annotated twofold peaks *S* along the crystallographic *x* axis (top) correspond to a packing peak of the two tetramers in the unit cell suggesting nearly (*i.e.* pseudo) orthorhombic symmetry.

Initial data processing, integration and data reduction at the beamline suggested that both crystals were orthorhombic (*P*222), with approximate unit-cell parameters *a* = 76, *b* = 101, *c* = 92 Å, or possibly monoclinic (*P*2), with a β angle of nearly 90° (range 89.56–90.06°) and with the unique twofold screw along the longest axis. The resolution limits of the data sets were in the range 2.4–2.7 Å. Further data-reduction analysis suggested that the data sets were all *P*2_1_, with various degrees of twinning that resulted in pseudo-orthorhombic symmetry that dominated the self-rotation function (SRF; Fig. 1 ▸). The degree of twinning was also assessed by examining the SRF (χ = 180° projection), which provided evidence for an approximately 222 symmetry tetramer in the asymmetric unit with one of the orthogonal twofold axes inclined a few degrees (7–10°) with respect to the crystallographic *c* axis (Fig. 1 ▸). A data set from crystal *B* extending to 2.4 Å resolution with refined unit-cell parameters *a* = 76.30, *b* = 100.17, *c* = 92.02 Å, β = 90.003° (*P*2_1_) was used for final refinement in combination with twinning corrections and estimated structure factors.

The size of the unit cell suggested that the asymmetric unit of the crystal contained four chains of *Ft*FBPaseII (328 amino acids plus the N-terminal His tag). In addition, the SRF provided evidence for three approximately orthogonal dyads related by the crystallographic 2_1_ screw axis. Given the high sequence identity to *Mt*FBPaseII (∼50%), a full tetramer was assembled from the dimer in the asymmetric unit of apo *Mt*FBPaseII (PDB entry 6ayu; Wolf *et al.*, 2018 ▸) and used as a model for molecular-replacement searches.

The earlier 2.7 Å resolution data set (with an unknown high twinning ratio) was used in the first attempts to solve the structure by molecular replacement. A suggestive solution was found, but subsequent refinement stalled at *R*_work_ = 0.30 and *R*_free_ = 0.42. Given the limited quality of the data and the electron-density maps, only part (60–70%) of the amino-acid sequence of the *Ft*FBPaseII protein could be fitted with confidence using these data. This model was subsequently used to solve and refine the structure with the best data set to 2.4 Å resolution (crystal *B*) using data with *I*/σ(*I*) > 3 (Table 1 ▸) to this resolution limit. Despite the significant twinning, this crystal allowed successful refinement after extensive model revisions for the different chains using the twinning option (refining against amplitudes) in *REFMAC*5 (Murshudov *et al.*, 2011 ▸). The presence of a twinning law and an estimation of the twin fraction (∼0.40) was also obtained using the *phenix.xtriage* module of *Phenix* (Liebschner *et al.*, 2019 ▸). The final refined twinning fractions were 0.574 and 0.426 for two twin domains, with twinning laws (*h*, *k*, *l*) and (−*h*, −*k*, *l*) or symmetry equivalents, respectively. Statistics for the data reduction for this data set are presented in Table 1 ▸, as well as the final refinement statistics.

The *CCP*4 suite of programs (Winn *et al.*, 2011 ▸), including *REFMAC*5 and *Coot* (Emsley *et al.*, 2010 ▸), was used for data analysis and refinement. In addition, 2–3 cycles of refinement using *Phenix* were typically also interspersed with the routine *REFMAC*5 refinement (ten cycles) to adjust the different scales and to consistently refine the twinning fraction. The extensive use of the *Phenix* refinement protocols resulted in the zeroing out of certain areas of the electron density in the most exterior loops that made it difficult to interpret them correctly. *REFMAC*5 protocols with no bulk-solvent correction were most effective in revealing the correct density for the external loops, most notably the insertion loop between Asn286 and Ser292, particularly in chain *A*. The interpretation of this loop in chain *A* was later built on the other three chains, where there was acceptable density. The same strategy was used to interpret the final 11 residues (Phe318–Ser328) of the chain, for which the density was only convincing in the final stages of the refinement (Supplementary Fig. S1). Previously, it had been interpreted as an additive in the crystallization medium.

*LSQKAB* (Kabsch, 1976 ▸) was used for structure analysis and superpositions, either using main-chain atoms or only C^α^ atoms. The superposition rotation matrices for the different pairs of subunits were used and the corresponding traces were used to extract the corresponding rotation angles. The resources of the *PDBsum* website were used to analyze the structure and compare the different quaternary structures (Laskowski, 2001 ▸; Laskowski *et al.*, 2018 ▸). *QtMG* (McNicholas *et al.*, 2011 ▸) was used to produce publication-quality figures.

## 3. Results   

### 3.1. Protein purification   

The purified protein was consistent in quality with that obtained previously for *Ft*FBPaseII (Gutka *et al.*, 2017 ▸). The quaternary structure of *Ft*FBPaseII is dependent on the protein concentration (Gutka *et al.*, 2017 ▸), with the tetrameric aggregate predominating in highly concentrated samples. The solubility of the purified wild-type and mutant proteins was high enough to allow the concentration of the samples to at least 10 mg ml^−1^. In order to increase the stability of the tetramer prior to experiments, all of the samples were handled and stored at concentrations of at least 10 mg ml^−1^, which was found to be critical for the reproducibility of the activity experiments.

### 3.2. Crystallization   

After the characterization of crystal *A*, a significant screening of crystallization conditions resulted in similar prismatic crystal forms (10–100 µm) but with etched surfaces. Crystals with the least amount of twinning and with diffraction data extending to the highest resolution were grown in 0.2 *M* sodium formate, 20% PEG 3350 in the presence of F6P (Supplementary Table S2). However, there were significant variations in the quality and extent of the different data sets from different crystals. Crystallographic analysis showed that they all belonged to the same monoclinic (*P*2_1_) space group with various degrees of twinning. In subsequent analysis, a data set from a crystal with the least amount of twinning was selected for the final refinement.

### 3.3. Quaternary structure   

The crystal structure of *Ft*FBPaseII revealed that an entire tetramer assembly (chains *A*–*D*) with approximate 222 (*D*_2_) symmetry was present in the asymmetric unit in the absence of any allosteric cofactors, giving support to the notion that this is the native oligomeric state under the crystallization conditions. Analysis of the corresponding inter-chain symmetry operations (Supplementary Table S3*a*) of the tetramer showed that the rotation angles ranged from 178.6° to 179.8° with negligible translational components.

This aggregation state has also been found in the asymmetric unit of the crystals of the *Synechocystis* dual enzyme (PDB entry 3rpl), in which the orthogonal symmetry operations were closer to exact twofold (179.7–179.9°) symmetry. This oligomeric arrangement is also consistent with the proposed tetramer in the structure of *Mt*FBPaseII created by the crystallographic twofold, duplicating the dimer (*AB*) in the asymmetric unit (Wolf *et al.*, 2018 ▸). The rotations that relate chains *A* and *B* in the asymmetric unit of native and T84S variant *Mt*FBPaseII are nearly the same, averaging 176.4° (Supplementary Table S3*b*). These values suggest minor differences among the orientation of the different subunits in the corresponding tetramers. Although there was only one monomer in the asymmetric unit for the first reported structure of the *E. coli* enzyme, a dimer was suggested in the initial report, and an exact 222 symmetry tetramer was subsequently suggested by the presence of a 222 symmetry center in the crystal lattice (Brown *et al.*, 2009 ▸; Wolf *et al.*, 2018 ▸).

**Figure 2:**
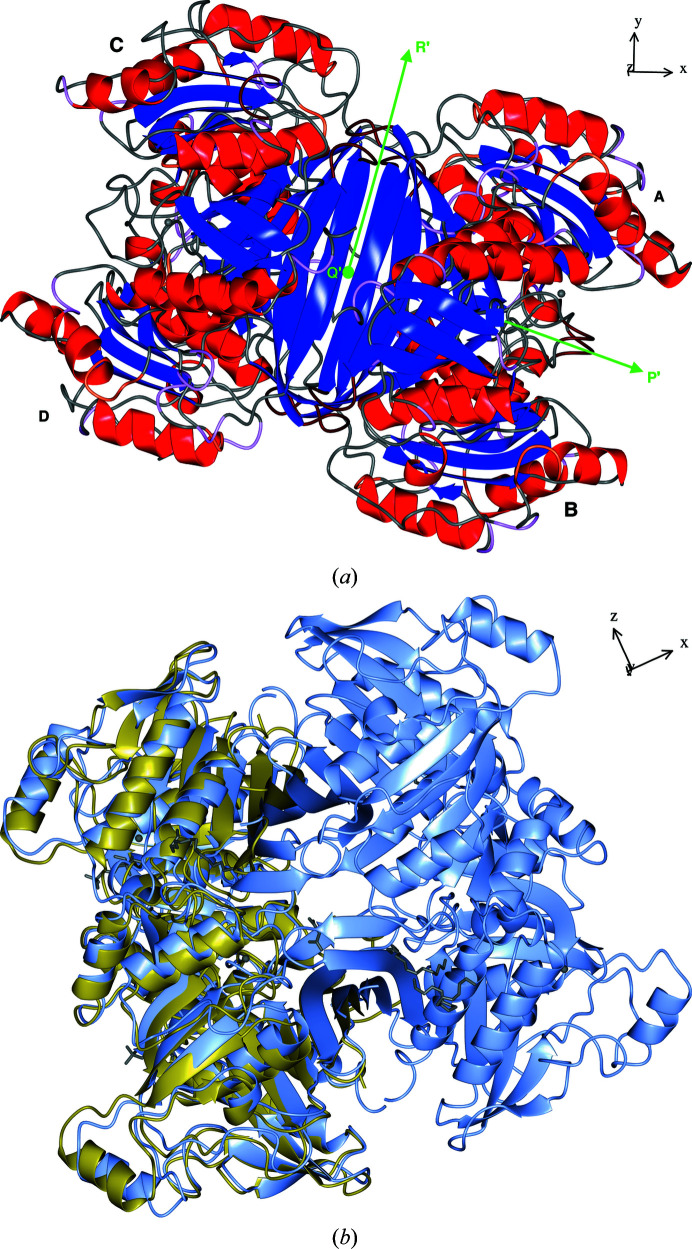
Structures of *Ft*FBPaseII and *Mt*FBPaseII. (*a*) A ribbon representation of the *Ft*FBPaseII tetramer is presented with the secondary-structure elements highlighted. Green arrows mark the directions of the three nearly orthogonal twofold axes relating the four chains *A*–*D*. The view is in the direction of the twofold axis corresponding to the dimer suggested for the structure of *Ec*FBPaseII (Brown *et al.*, 2009 ▸), which contains only one chain in the asymmetric unit. The nearly horizontal twofold (∼15° off) is the same as that found in the asymmetric unit of *Mt*FBPaseII (Wolf *et al.*, 2018 ▸). (*b*) An overall superposition of the dimer of *Mt*FBPaseII (PDB entry 6ayu) in the asymmetric unit on the tetramer of *Ft*FBPaseII is presented. The direction of the crystallographic axes in the corresponding views is shown on the upper right and can be related to the SRF function results illustrated in Fig. 1 ▸. The chains in (*a*) are labeled with different font sizes to provide a sense of perspective: larger sizes are in the foreground. The view in (*b*) emphasizes the protruding helix feature (H12) that extends from the body of the tetramer and is prominent in this enzyme class (see the sequence alignment in Fig. 3 ▸).

A quantitative comparison of the tetramer interfaces in the structure of *Ft*FBPaseII with those in the structure with PDB code 3rpl, which also contains a full tetramer in the asymmetric unit of the hexagonal lattice (*P*6_5_; Table 2 ▸), reveals a consistent pattern of interactions. The equivalent interfaces (*A*–*D* and *B*–*C*) in the two oligomers are formed by the largest number of hydrogen bonds, although the *Ft*FBPaseII tetramer contains more polar interactions. This is the dimer interface (*A*–*D*) that was initially highlighted in the structure of *Ec*FBPaseII, where the contact surface is dominated by two long antiparallel β-strands forming an extended β-sheet (Fig. 2 ▸ *a*) perpendicular to the dyad. The other two equivalent interfaces (*A*–*B* and *C*–*D*) were revealed as important in the asymmetric unit of *Mt*FBPaseII (Wolf *et al.*, 2018 ▸), with a rather large number of nonbonded contacts in PDB entry 3rpl (184–195) and approximately the same contacts in *Ft*FBPaseII (187–189), with an additional three salt bridges (Table 2 ▸).

### 3.4. Three-dimensional structure   

Given the different crystallographic environments of the four chains in the asymmetric unit, the quality of the tertiary structures of the different chains (*A*–*D*) varies significantly. In the proximity of the active-site region, the presence or absence of Mg^2+^ cations makes an appreciable difference in ordering the amino-acid side chains in their proximity. Chains *A* and *B* are the best defined in terms of electron density and structural quality, followed by *D* and *C*, respectively. The numbers of Ramachandran outliers are 0, 2, 3 and 3, respectively.

**Figure 3:**
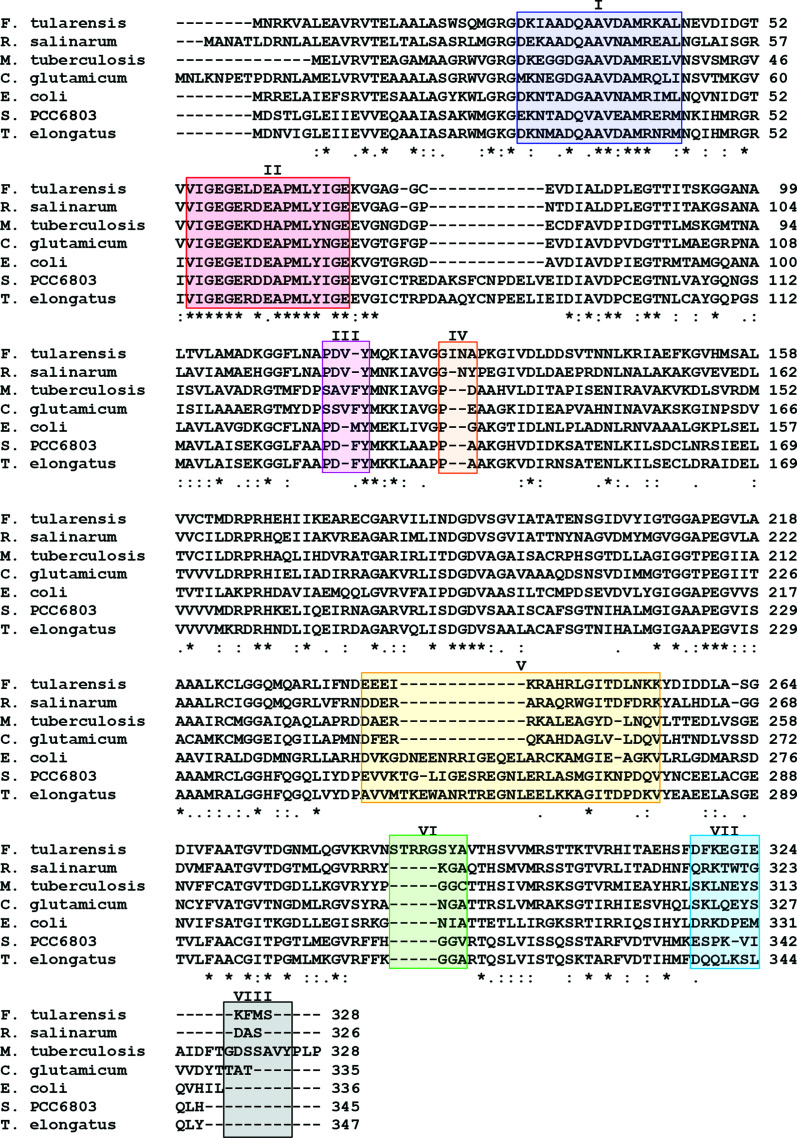
Amino-acid sequence alignment of *Ft*FBPaseII with other closely related class II FBPases. The overall amino-acid sequence alignment of the most closely related class II FBPases is presented. The highlighted regions annotated with Roman numerals (I–VIII) correspond to the areas where chains *A* and *D* differ the most structurally (Figs. 4 ▸ *a* and 4 ▸ *b*). The amino-acid sequence of *Ft*FBPaseII often deviates from the other related class II FBPases near these regions.

Chain *A* of *Ft*FBPaseII definitively establishes the details of the secondary structure and the tertiary fold of the *Ft*FBPaseII enzyme as a five-layer α/β sandwich very similar to the structures of *Ec*FBPaseII (PDB entry 3d1r) and *Mt*FBPaseII (PDB entries 6ayy, 6ayu and 6ayv) and the *Synechocystis* dual enzyme (PDB entry 3rpl), although distinct features are also present. The secondary-structure analysis reveals the presence of 14 α-helices and 14 β-strands of different lengths organized into two nearly orthogonal β-sheets (sheets *A* snd *B*; Supplementary Fig. S2). A significant component of β-sheet *A* is a distinct ψ-loop comprising residues Asp48–Asp84 that forms the first three most external β-strands; a similar feature is also present in β-sheet *B* but is not as prominent or well defined. These external strands of sheet *A* have the highest temperature factors of all of the residues in chains *A* and *B* and are not as well defined in chains *C* and *D*. The insertions and deletions suggested by the amino-acid sequence alignment are typically accommodated outside the secondary-structural elements by ordered loops and connecting fragments (Fig. 3 ▸).

**Figure 4:**
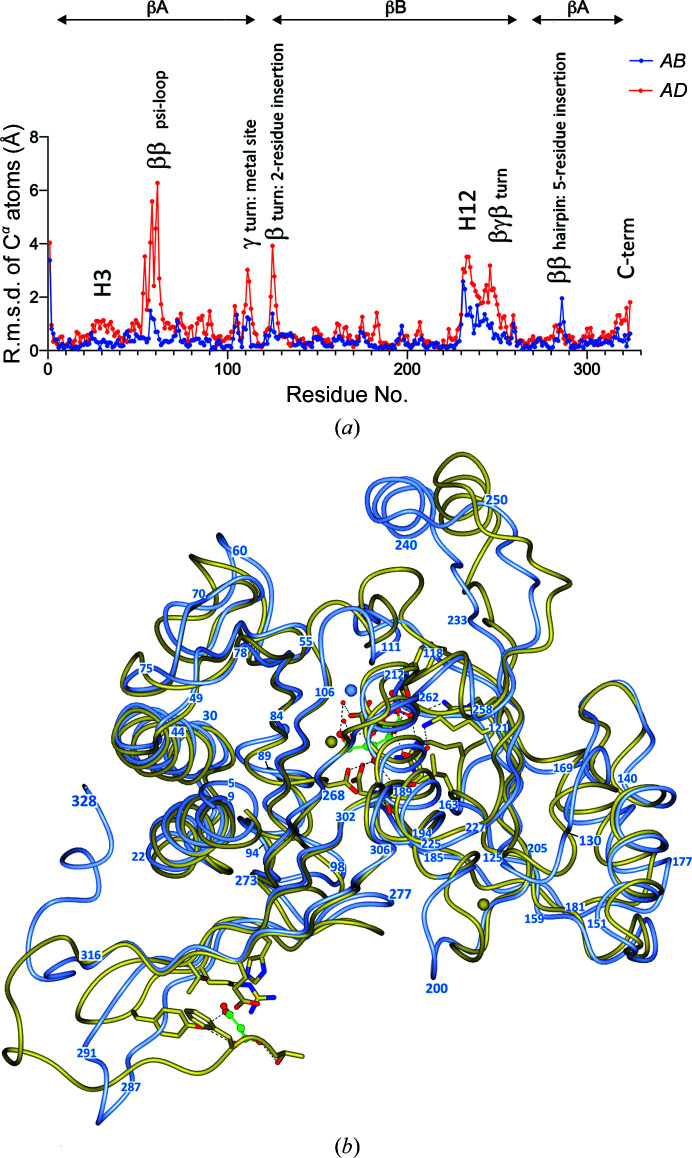
(*a*) There are significant differences between the three-dimensional structures of the four chains (Supplementary Table S3) in the *Ft*FBPaseII tetramer. Blue: the r.m.s.d.s versus residue number for chains *A* and *B* are relatively small. Red: in contrast, the r.m.s.d.s versus residue number for chains *A* and *D* are larger, particularly in the loops connecting the β-­strands of the ψ-loop (residues 48–70; Supplementary Table S3). The deviations observed between the two pairs (*A*/*B* and *A*/*D*) highlight the most flexible areas of the structure and suggest that the observed differences occur in the same regions but are larger between the *A* and *D* chains. (*b*) Superposition of the structure of *Ft*FBPaseII (chain *A*, blue) with the corresponding chain of *Mt*FBPaseII (PDB entry 6ayu, gold). The C^α^–C^α^ coil superposition is shown in an orientation highlighting the regions of most significant structural difference, which correspond to regions of insertions/deletions in the amino-acid sequence (Fig. 3 ▸). The course of the polypeptide chain has been annotated with numbers that can be related to the regions with large r.m.s.d.s in (*a*). Significant regions are the differences in the protruding helix extension (top right) and the carboxy end (lower left) (see Supplementary Fig. S4 for further detail). The active site in *Mt*FBPaseII contains the product and related atoms for reference and also a malonate molecule near the carboxy end.

There are a few noteworthy single amino-acid differences between species (Fig. 3 ▸). Firstly, one residue is deleted in *Ft*FBPaseII (corresponding to Asp70 in *Mt*FBPaseII and Arg76 in *Ec*FBPaseII) in the structurally conserved ψ-loop that ends by projecting the first metal-binding residue Asp84. Secondly, there is an insertion in *Mt*FBPaseII of Phe113 prior to the catalytically important Tyr114 (Tyr119 in *Ft*FBPase), which results in a local distortion prior to the catalytically conserved residue Tyr119. The insertion results in the extension in *Ft*FBPaseII and *Ec*FBPaseII of an aspartic acid residue (Asp116) into the metal cation site (see below; Fig. 3 ▸). Thirdly, a two-residue insertion is present in *Ft*FBPaseII after Gly128 (Ile129-Asn130), which extends and bulges out towards the five-residue insertion (Ser287-Thr288-Arg289-Arg290-Gly291) between Asn286 and Ser292, and critically interacts with these residues in chain *D* of the tetramer (Fig. 3 ▸). The r.m.s.d.s between the C^α^ atoms of chains *A* and *B* and chains *C* and *D* of the tetramer are shown in Fig. 4 ▸(*a*). Finally, the C-terminal 11 amino acids adopt a unique fold forming a compact hydrophobic core that includes three Phe residues (Phe317, Phe319 and Phe326) and a short helical segment (H14), packing against Trp21 (Supplementary Fig. S1), that is unique to *Ft*FBPase. *BLAST* amino-acid sequence searches of the final 11-residue sequence revealed that the sequence is highly conserved (>90%) in all species of the *Francisella* genus. The function of this conserved structural feature is unknown.

### 3.5. Comparison with other related class II FBPases   

The most significant differences among the three closely related structures (*Ec*FBPaseII, *Mt*FBPaseII and *Ft*FBPaseII) are in the loops extending towards the protruding helix (H12) and the return loops connecting back towards the core of the tetramer structure. These connecting loops are longer in *Ec*FBPaseII and far shorter in the other two structures. Nonetheless, a 13-residue helical stretch (Asp237–Gly249) in *Ft*FBPaseII superimposes in register with Gly248–Gly260 in *Ec*FBPaseII. This helical stretch is significantly displaced in *Mt*FBPaseII because of the presence of a two-residue insertion prior to Tyr119 in *Mt*FBPaseII that bulges out of the otherwise regular β-strand in *Ft*FBPaseII (Figs. 3 ▸, 4 ▸ *a* and 4 ▸ *b*). The functional significance of these helical protrusions is as yet unknown. The r.m.s.d.s between chain *A* of *Ft*FBPaseII and the corresponding chain *A* of its closest amino-acid sequence homolog (*Mt*FBPaseII) are shown in Fig. 4 ▸(*b*). The regions showing significant differences are annotated in Fig. 4 ▸(*a*) within the context of the overall α/β fold.

There are also noteworthy three-dimensional structure alterations in *Ft*FBPaseII induced by the concomitant insertions after Gly126 (Ile127-Asn128) and after Asn286 (Ser287-Thr288-Arg289-Arg290-Gly291) (Figs. 3 ▸, 4 ▸ *a* and 4 ▸ *b*). Intriguingly, the two dominant polar residues of the insertion, Arg289-Arg290, are not exposed to the surface and interact rather significantly with the cluster of side chains and C=O groups of Thr198, Ser201, Ile203 and Asp204 (chain *A*–chain *D* interface). Alternatively, Arg290 interacts with the C=O group of Leu225 in chain *D* and the side chain of Glu314 in chain *A*. In addition to reinforcing the inter-chain interactions on the periphery of the tetramer by the noncrystallographic symmetry, the resulting two longer β-strands (Met278–Thr288 and Arg290–Ser303) and the connecting hairpin loop curve in a concave fashion to create a medium-sized pocket inside the tetramer that corresponds to the pocket where the allosteric effector AMP is found in the *Synechocystis* dual enzyme (PDB entry 3rpl). Intriguingly, this confirmed allosteric pocket is instead formed by an extension of the C-terminus, which is about 20 amino acids longer in PDB entry 3rpl.

Finally, the last 12 residues at the carboxy end of the *Ft*FBPaseII tetramer fold form a compact hydrophobic core dominated by the presence of three Phe residues (Phe317, Phe319 and Phe326) packing next to Trp21. This conformation differs from that observed in *Mt*FBPaseII and is significantly different in the *Synechocystis* dual enzyme, where it is found bound to the allosteric effector AMP in a well defined pocket (Feng *et al.*, 2014 ▸).

### 3.6. Enzymatic analysis of *Ft*FBPaseII activity   

**Figure 5:**
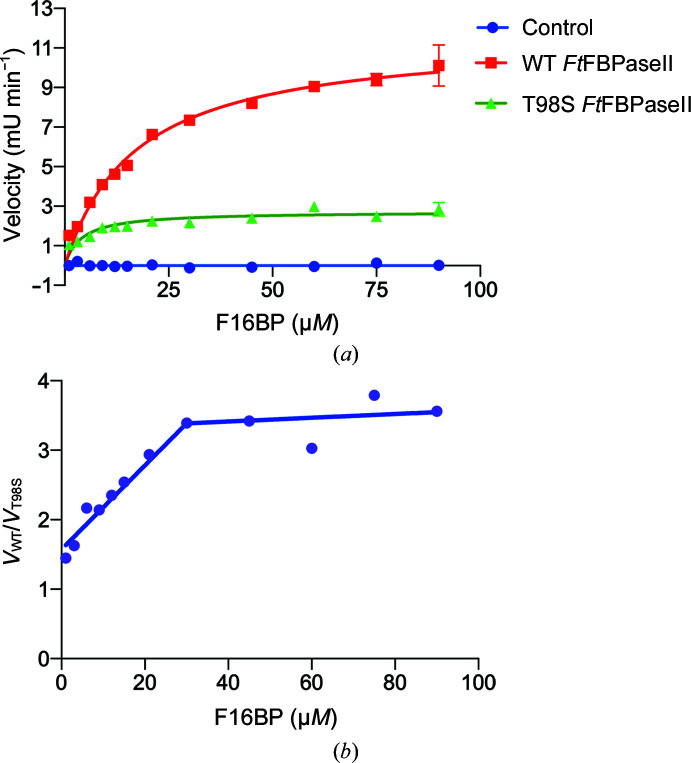
Enzymatic activity of native active-site mutants of *Ft*FBPase. (*a*) The enzymatic activity of native *Ft*FBPaseII and its T89S and T89A mutants versus F16BP (substrate) concentration. The T89S mutant has a lower *V*_max_ than the wild type by approximately fourfold (Table 3 ▸), while the T89A mutant is inactive. (*b*) Plot of the ratio of the rates for the wild type and T89S mutant (*V*_WT_/*V*_T89S_) as a function of substrate concentration (F16BP). This type of analysis has been suggested (Eisenthal *et al.*, 2007 ▸) to compare the activity of two enzyme variants (wild type and mutants) catalyzing the same reaction in more detail. The substrate concentration has to reach a level above the *K*_m_ for both enzymes to show a constant ratio between the two enzyme rates.

The experimentally determined reaction velocity plotted against concentration of substrate for both wild-type and T89S variant *Ft*FBPaseII exhibited a typical Michaelis–Menten response (Fig. 5 ▸ *a*, Table 3 ▸). The *K*_m_ of wild-type *Ft*FBPaseII was calculated to be 17 ± 4 µ*M*, which is in agreement with previously published observations for coupled assays with real-time measurements (Gutka *et al.*, 2017 ▸; Supplementary Table S4 and Fig. S3). The *K*_m_ for the T89S variant was determined to be 4 ± 2.1 µ*M*, which is approximately four times lower than that of the wild-type enzyme.

Thus, wild-type *Ft*FBPaseII needs to reach a substrate concentration of 17 ± 4 µ*M* to reach half of its maximal velocity (0.9 µmol min^−1^ mg^−1^); this value is nearly five times higher than that for *Mt*FBPaseII. In contrast, the T89S mutant requires about a fourfold lower substrate concentration to achieve half of its maximal velocity, which is also approximately four times lower (0.2 µmol min^−1^ mg^−1^). The turnover rate of the T89S mutant is also approximately four times lower than that of the wild type. While the wild-type enzyme takes approximately 2 s to convert one substrate molecule into product, the T89S mutant takes approximately four times longer (8 s). The *k*_cat_/*K*_m_ values are approximately the same (33 and 31 s^−1^ m*M* ^−1^, respectively) for the wild type and the T89S variant.

In view of the ambiguities and inconsistencies in using *k*_cat_/*K*_m_ to compare the activities of different enzymes (mutants) for the same substrate (Eisenthal *et al.*, 2007 ▸), we instead used the ratio of the rate of the two enzymes (*V*_WT_/*V*_T89S_) as a function of substrate concentration. The results are shown in Fig. 5 ▸(*b*). The analysis indicates that the ratio of the rates of the two enzymes increases linearly from 1.5 when the concentration of substrate is below or near the *K*_m_ values of the enzymes, reaching a constant value of nearly 3.5 when the enzymes reach their maximum velocities (Fig. 5 ▸ *b*).

### 3.7. Active-site environment   

The best-defined side-chain residues within the active-site structure are observed in chain *A*, where two Mg^2+^ sites contribute to stabilizing acidic residues. However, in the crystal structure this active-site region, in chain *A* only, is essentially covered by residues from the symmetrically related chain *D*. This packing effect results in an active site with no space left for the substrate/product (F16BP/F6P) that was present in the crystallization medium. Moreover, two residues from the symmetry-related chain *D* (Glu238 and Glu239) make distinct interactions with residues in the active site which, although providing a more rigid environment, are likely to affect the resulting structure by their presence.

**Figure 6:**
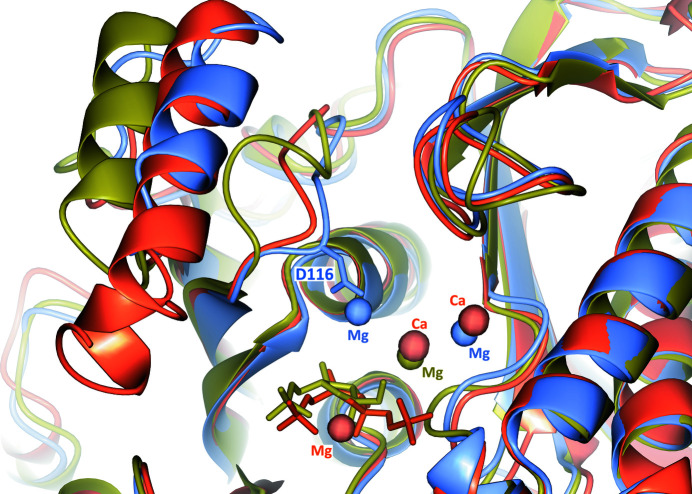
Superposition of the metal sites in *Ec*FBPaseII, *Ft*FBPaseII and *Mt*FBPaseII. The β-bulge in the structure of *Mt*FBPaseII (gold; PDB entry 6ayu) pushes out the ‘protruding’ helix and displaces it with respect to the two aligned helices of *Ec*FBPaseII (PDB entry 3d1r; red) and *Ft*FBPaseII (this work; blue). Metal cation positions are shown as spheres of different colors corresponding to the proteins (*Ec*FBPaseII, two Ca^2+^ and Mg^2+^ in red and substrate F16BP; *Ft*FBPaseII, two Mg^2+^ in blue in chain *A*; MtFBPaseII, one Mg^2+^ in gold next to the product F6P). The Mg^2+^ site on the left (near Asp116) is unique. The extended loop contains Asp116 (side chain shown) as a ligand of the unique Mg^2+^ site found in this structure of *Ft*FBPaseII (see Fig. 7 ▸). The superposition shows that this Mg^2+^ site is distant (∼7.4 Å) from the cleavable/leaving phosphate, suggesting that such a metal site is not catalytically relevant but probably inhibits the enzyme.

Glu238 is positioned approximately between Mg^2+^(1) and Mg^2+^(2). The first cation is nearly octahedrally coordinated by the side chain of Asp84 and the the C=O group of Leu86 plus four water molecules. The second cation, Mg^2+^(2), interacts weakly in an approximately trigonal bipyramid coordination with the side chains of Glu214 and Asp116 and two water molecules at the apices of the pyramid. Asp116 extends from the main chain as a result of the sequence differences between *Ft*FBPaseII and *Mt*FBPaseII prior to the conserved Tyr118 (Asp116-Val117-Tyr118; Fig. 3 ▸). Superposition of the active sites of *Ec*FBPaseII, *Mt*FBPaseII and *Ft*FBPaseII (chain *A*) (Fig. 6 ▸) reveals that the second Mg^2+^ site is unique to this structure and is distant (∼7.4 Å) from the cleavable phosphate group of the substrate. Thus, such a site would probably not participate in the catalytic reaction but could result in enzyme inhibition owing to its interaction with Glu214 that is normally liganded to the conserved Mg^2+^/Ca^2+^ site in the structures of *MtF*BPaseII (PDB entry 6ayu) and *Ec*FBPaseII (PDB entry 3d1r) (Fig. 6 ▸).

An additional packing interaction between chain *A* (Arg165-Pro166-Arg167) and Asp237 from the crystallo­graphically related chain *D* places its carboxylate side chain in a position and orientation similar (∼1 Å) to two phosphate O atoms of the product F6P, as observed in the *Mt*FBPaseII–F6P (PDB entry 6ayu) complex. The two arginine residues adopt a similar conformation, as their guanidium groups are found to be positioned within 1.5 Å (1.2 and 1.5 Å) of the corresponding groups in *Mt*FBPaseII (Arg159-Pro160-Arg161). Most likely owing to the alteration in the active site of the Mg^2+^-inhibited enzyme, the substrate-binding site was un­occupied, and these interactions formed instead of the normal binding of F16BP/F6P, even though the former was present in the crystallization medium at a 1 m*M* concentration.

## 4. Discussion   

Protein purification using size-exclusion chromatography suggested the presence of both dimers and tetramers in solution, which was confirmed by the presence of a tetramer in the asymmetric unit of the *Ft*FBPaseII crystals. Distinct amino-acid insertions and deletions in the polypeptide chain of *Ft*FBPaseII seem to strengthen the interactions among the four chains of the *A*–*D* oligomer, resulting in a stable, approximately 222 symmetry tetramer for the enzymatic assembly of *Ft*FBPase. At the concentrations used to grow the crystals, the tetramer is most likely to be the predominant species. The tetramer interfaces are conserved in the different enzymes characterized thus far, although the relative numbers of hydrogen bonds, salt bridges and nonbonded contacts vary among the residues involved. The two structures with a full tetramer in the asymmetric unit, the *Synechocystis* dual enzyme (PDB entry 3rpl) and *Ft*FBPaseII, have similar numbers of residues participating in the interfaces, residues 26–30 and 22–36, respectively, although the latter has nearly double the number of salt bridges. The structure reported in this work is consistent with the previous suggestion that the native conformation of this class of enzymes is tetrameric. Whether this association implies the existence of allosteric regulators, as is the case for PDB entry 3rpl, remains un­confirmed. Confirming the existence of allosteric effectors and their binding pockets would be very important for further inhibitor design against this important class of enzymes.

The persistent presence of various degrees of twinning and our failure to grow genuine single crystals of this enzyme could be related to the approximate 222 symmetry of the tetramer, whereby tetramers with slightly different orientations along the crystal cell axes could result in twinning domains and/or disorder throughout a low-symmetry crystal lattice such as *P*2_1_.

The existence of strategic insertions and deletions along the amino-acid sequence of *Ft*FBPaseII, combined with the observed local three-dimensional structural alterations along the polypeptide chain near Tyr119, may provide a rationale for the different cation dependence, with Mg^2+^, Mn^2+^, Zn^2+^ and Li^+^ sensitivity being observed for the various members of this class. Unfortunately, our crystals grew under crystallization conditions that did not contain Mn^2+^, the putative genuine metal cofactor of *Ft*FBPaseII and *Ec*FBPaseII. Instead, Mg^2+^ was found in the corresponding active site. In chain *A*, our best-defined monomer structure, two Mg^2+^ sites were found. Mg^2+^(1) was near a Ca^2+^ site in *Ec*FBPaseII with the corresponding conserved ligands. The presence of a second site Mg^2+^(2) next to it and interacting with Asp116, a unique ligand extending from the altered structure of the chain next to Tyr118, was observed. Asp116 in *Ft*FBPaseII is also conserved in *Ec*FBPaseII (Asp117) and superimposes within 1.3 Å (C^α^–C^α^ distance) with the same side-chain orientation. Previous biochemical and enzymatic characterization of *Ft*FBPaseII (Gutka *et al.*, 2017 ▸) has shown that the enzyme is only active in the presence of Mn^2+^, as opposed to other divalent (Zn^2+^, Cu^2+^ and Ni^2+^) or monovalent (K^+^ and Na^+^) cations. In addition, FBPase class II enzymes that depend exclusively on Mn^2+^ for activity have also been found to be inhibited by Mg^2+^ (Gutka *et al.*, 2017 ▸). Thus, it is reasonable to hypothesize that the crystal structure of *Ft*FBPaseII presented here, and in particular the presence of the second Mg^2+^ site (above), corresponds to the Mg^2+^-inhibited structure, most likely owing to the presence of Mg^2+^ in nonproductive metal complexes instead of the catalytic Mn^2+^.

Initial enzymatic and kinetic studies of two *Ft*FBPaseII protein variants at the active-site residue Thr89 (T89S and T89A) showed that the former is partially active while the latter is inactive. This observation is consistent with similar results for the *Mt*FBPaseII enzyme (Bondoc *et al.*, 2017 ▸). The observed reduction in the activity of the T89S mutant corresponds to an approximate fourfold reduction in the *V*_max_ value and a similar reduction in the turnover number (*k*_cat_). The previously published structures of wild-type and T84S variant (with an equivalent Thr residue) *Mt*FBPaseII complexed with the product F6P (PDB entries 6ayu and 6ayv, respectively; Wolf *et al.*, 2018 ▸) do not show any significant differences within an 8 Å radius of the bound substrate. Thus, it is likely that the binding pockets for the substrate are unaltered between the two enzyme forms. Consequently, assuming that the hypothetical dissociation constant *K*_d_ (where *K*_d_ = *k*_−1_/*k*_1_) for the substrate is essentially unchanged by the replacement of Thr89 by Ser89 in the variant enzyme, we hypothesize that the amino-acid replacement results in an enzyme that is approximately four times slower in turning over substrate into product. The detailed structural explanations of this observation are unknown, mainly because the structure of *Ft*FBPaseII discussed does not represent an active enzyme with the native conformation of the Mn^2+^ metal cofactor and with substrate (or product) present.

In summary, the crystallographic structure of *Ft*FBPaseII is presented. The structure reveals an approximately 222 symmetry tetramer in the asymmetric unit that exhibits subtle structural alterations in some peripheral loops, resulting from amino-acid residue insertions, that probably stabilize the tetrameric structure. In addition, the last 11 residues of the polypeptide adopt a compact globular structure that also contributes to the stability of the aggregate. In contrast, in other class II FBPases the tetramer is generated partially or fully by the presence of crystallographic symmetry (in *Mt*FBPaseII and *Ec*FBPaseII, respectively). It is uncertain whether the approximately tetrameric structure is owing to the lack of a still unknown allosteric effector. Other significant alterations were observed prior to the catalytically important Tyr118 that resulted in the projection of Asp116 into a metal ligand pocket where an Mg^2+^ cation was found. This cation site does not correspond to any of the sites present in other class II FBPases for which the structures are known. The suggestion is made that the present structure corresponds to an Mg^2+^-inhibited FBPase. Future experiments are being planned to confirm the functional importance of this structural alteration near the binding pocket of the substrate and its importance for cation specificity in class II FBPases. In addition, further enzymatic and kinetic studies will be pursued in the search for possible allosteric effectors of this class of enzymes.

## Supplementary Material

PDB reference: class II fructose-1,6-bisphosphatase from *F. tularensis*, 7js3

Supplementary Figures and Tables. DOI: 10.1107/S2053230X20013370/dw5213sup1.pdf

## Figures and Tables

**Figure 7:**
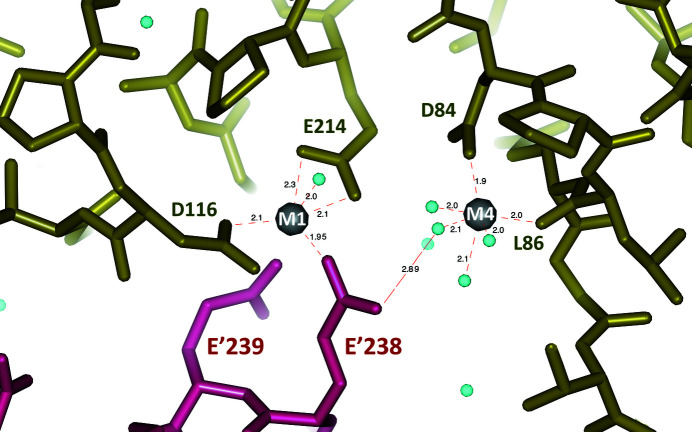
*Ft*FBPaseII metal sites as found in chain *A* of the *Ft*FBPaseII tetramer. The right (M4) site has a more conventional octahedral coordination by water molecules plus the side chain of Asp84 (chain *A*) and the carbonyl group of Leu86 (chain *A*). On the left is the M1 site created by the extension of Asp116 in *Ft*FBPaseII relative to *Mt*FBPaseII prior to Tyr118. At the bottom, the side chain of Glu328 from the symmetrically related molecule (chain *D*; purple) bridges the two metal sites. In this structure, the M1 site appears to be unique to this group of class II FBPases and probably corresponds to an Mg^2+^-inhibited form of the enzyme. *Ft*FBPaseII uses Mn^2+^ as a metal cofactor for activity.

**Table 1:** Data-collection and refinement statistics for *Ft*FBPaseII (PDB entry 7js3) The data-reduction and refinement variables have their standard definitions. Values in parentheses are the highest resolution shell.

Wavelength (Å)	0.97856
Resolution (Å)	40.0–2.40 (2.44–2.40)
Space group	*P*2_1_
*a*, *b*, *c* (Å)	76.30, 100.17, 92.02
α, β, γ (°)	90, 90.003, 90
Total reflections	196190
Unique reflections	53682 (2620)
Multiplicity	3.6 (3.7)
Completeness (%)	98.9 (97.5)
Mean *I*/σ(*I*)	24.6 (3.2)
Wilson *B* factor (Å^2^)	42.9
*R*_meas_	0.106 (0.531)
CC_1/2_	0.991 (0.673)
*R*_p.i.m._	0.055 (0.276)
Twinning fraction	0.432
Twinning operator	*h*, −*k*, −*l*
Reflections used in refinement	53626 (2502)
Reflections used for *R*_free_	2732 (119)
*R*_work_	0.154 (0.185)
*R*_free_	0.193 (0.292)
No. of non-H atoms	
Total	9734
Macromolecules	9434
Ligands	4
Solvent	269
No. of protein residues	1268
R.m.s.d. from ideal	
Bond lengths (Å)	0.010
Angles (°)	1.24
Ramachandran plot	
Favored (%)	92.0
Allowed (%)	7.6
Outliers (%)	0.6
Clashscore	15
Average *B* factor (Å^2^)	
Overall	52.6
Macromolecule	52.7
Ligands	71.9
Solvent	44.9

**Table 2:** Tetramer interfaces

Chains	No. of residues	Interface area† (Å^2^)	No. of salt bridges	No. of hydrogen bonds	No. of nonbonded contacts
PDB entry 3rpl					
*A*–*B*	27:26	1320:1300	—	16	184
*C*–*D*	26:27	1365:1340	—	19	195
*A*–*D*	28:26	1580:1580	3	20	147
*B*–*C*	30:27	1610:1600	3	20	154
*A*–*C*	1:1	36:36	—	—	1
*B*–*D*	1:1	34:36	—	—	1
*Ft*FBPase					
*A*–*B*	30:30	1480:1500	3	21	187
*C*–*D*	31:29	1460:1460	3	16	189
*A*–*D*	24:33	1480:1410	5	23	157
*B*–*C*	25:33	1330:1380	5	19	149
*A*–*C* ‡	2:2	110:110	—	1	4
*B*–*D* ‡	1:1	80:80	—	—	2

† Interface area figures have been rounded to their last significant values.

‡ Interfaces *A*–*C* and *B*–*D* are not considered equivalent according to the *PDBsum* protein–protein interaction analysis software.

**Table 3:** Kinetic parameters for wild-type and T89S mutant *Ft*FBPaseII

*Ft*FBPaseII	*K*_m_ (µ*M*)	*V*_max_ (µmol min^−1^ mg^−1^)	*k*_cat_ (s^−1^)	*k*_cat_/*K*_m_ (s^−1^ m*M* ^−1^)
Wild type	17 ± 4	0.9 ± 0.02	0.55 ± 0.04	33
T89S	4 ± 2.1	0.2 ± 0.03	0.12 ± 0.02	31

## References

[bb1] Bondoc, J. M. G., Wolf, N. M., Ndichuck, M., Abad-Zapatero, C. & Movahedzadeh, F. (2017). *Biotechnol. Rep.* **15**, 48–54. 10.1016/j.btre.2017.06.004 PMC5485559 28702369

[bb2] Brissac, T., Ziveri, J., Ramond, E., Tros, F., Kock, S., Dupuis, M., Brillet, M., Barel, M., Peyriga, L., Cahoreau, E. & Charbit, A. (2015). *Mol. Microbiol.* **98**, 518–534. 10.1111/mmi.13139 26192619

[bb3] Brown, G., Singer, A., Lunin, V. V., Proudfoot, M., Skarina, T., Flick, R., Kochinyan, S., Sanishvili, R., Joachimiak, A., Edwards, A. M., Savchenko, A. & Yakunin, A. F. (2009). *J. Biol. Chem.* **284**, 3784–3792. 10.1074/jbc.M808186200 PMC2635049 19073594

[bb4] Dennis, D. T., Inglesby, T. V., Henderson, D. A., Bartlett, J. G., Ascher, M. S., Eitzen, E., Fine, A. D., Friedlander, A. M., Hauer, J., Layton, M., Lillibridge, S. R., McDade, J. E., Osterholm, M. T., O’Toole, T., Parker, G., Perl, T. M., Russell, P. K. & Tonat, K. (2001). *JAMA*, **285**, 2763–2773. 10.1001/jama.285.21.2763 11386933

[bb5] Eisenthal, R., Danson, M. J. & Hough, D. W. (2007). *Trends Biotechnol.* **25**, 247–249. 10.1016/j.tibtech.2007.03.010 17433847

[bb96] Emsley, P., Lohkamp, B., Scott, W. G. & Cowtan, K. (2010). *Acta Cryst.* D**66**, 486–501. 10.1107/S0907444910007493 PMC2852313 20383002

[bb6] Feng, L., Sun, Y., Deng, H., Li, D., Wan, J., Wang, X., Wang, W., Liao, X., Ren, Y. & Hu, X. (2014). *FEBS J.* **281**, 916–926. 10.1111/febs.12657 24286336

[bb8] Guryčová, D. (1998). *Eur. J. Epidemiol.* **14**, 797–802. 10.1023/a:1007537405242 9928875

[bb9] Gutka, H. J., Rukseree, K., Wheeler, P. R., Franzblau, S. G. & Movahedzadeh, F. (2011). *Appl. Biochem. Biotechnol.* **164**, 1376–1389. 10.1007/s12010-011-9219-x 21451980

[bb10] Gutka, H. J., Wolf, N. M., Bondoc, J. M. G. & Movahedzadeh, F. (2017). *Appl. Biochem. Biotechnol.* **183**, 1439–1454. 10.1007/s12010-017-2512-6 PMC5698383 28547120

[bb85] Kabsch, W. (1976). *Acta Cryst.* A**32**, 922–923.

[bb11] Kadzhaev, K., Zingmark, C., Golovliov, I., Bolanowski, M., Shen, H., Conlan, W. & Sjöstedt, A. (2009). *PLoS One*, **4**, e5463. 10.1371/journal.pone.0005463 PMC2675058 19424499

[bb12] Keim, P., Johansson, A. & Wagner, D. M. (2007). *Ann. N. Y. Acad. Sci.* **1105**, 30–66. 10.1196/annals.1409.011 17435120

[bb13] Laskowski, R. A. (2001). *Nucleic Acids Res.* **29**, 221–222. 10.1093/nar/29.1.221 PMC29784 11125097

[bb90] Laskowski, R. A., Jabłońska, J., Pravda, L., Vařeková, R. S. & Thornton, J. M. (2018). *Protein Sci.* **27**, 129–134. 10.1002/pro.3289 PMC5734310 28875543

[bb98] Liebschner, D., Afonine, P. V., Baker, M. L., Bunkóczi, G., Chen, V. B., Croll, T. I., Hintze, B., Hung, L.-W., Jain, S., McCoy, A. J., Moriarty, N. W., Oeffner, R. D., Poon, B. K., Prisant, M. G., Read, R. J., Richardson, J. S., Richardson, D. C., Sammito, M. D., Sobolev, O. V., Stockwell, D. H., Terwilliger, T. C., Urzhumtsev, A. G., Videau, L. L., Williams, C. J. & Adams, P. D. (2019). *Acta Cryst.* D**75**, 861–877. 10.1107/S2059798319011471 PMC6778852 31588918

[bb7] McNicholas, S., Potterton, E., Wilson, K. S. & Noble, M. E. M. (2011). *Acta Cryst.* D**67**, 386–394. 10.1107/S0907444911007281 PMC3069754 21460457

[bb99] Murshudov, G. N., Skubák, P., Lebedev, A. A., Pannu, N. S., Steiner, R. A., Nicholls, R. A., Winn, M. D., Long, F. & Vagin, A. A. (2011). *Acta Cryst.* D**67**, 355–367. 10.1107/S0907444911001314 PMC3069751 21460454

[bb14] Otwinowski, Z. & Minor, W. (1997). *Methods Enzymol.* **276**, 307–326. 10.1016/S0076-6879(97)76066-X 27754618

[bb15] Radlinski, L. C., Brunton, J., Steele, S., Taft-Benz, S. & Kawula, T. H. (2018). *mBio*, **9**, e01471-18. 10.1128/mBio.01471-18 PMC6247087 30459188

[bb16] Rittmann, D., Schaffer, S., Wendisch, V. F. & Sahm, H. (2003). *Arch. Microbiol.* **180**, 285–292. 10.1007/s00203-003-0588-6 12904832

[bb17] Santic, M., Molmeret, M., Klose, K. E. & Abu Kwaik, Y. (2006). *Trends Microbiol.* **14**, 37–44. 10.1016/j.tim.2005.11.008 16356719

[bb18] Sjöstedt, A. (2006). *Microbes Infect.* **8**, 561–567. 10.1016/j.micinf.2005.08.001 16239121

[bb97] Winn, M. D., Ballard, C. C., Cowtan, K. D., Dodson, E. J., Emsley, P., Evans, P. R., Keegan, R. M., Krissinel, E. B., Leslie, A. G. W., McCoy, A., McNicholas, S. J., Murshudov, G. N., Pannu, N. S., Potterton, E. A., Powell, H. R., Read, R. J., Vagin, A. & Wilson, K. S. (2011). *Acta Cryst.* D**67**, 235–242. 10.1107/S0907444910045749 PMC3069738 21460441

[bb19] Wolf, N. M., Gutka, H. J., Movahedzadeh, F. & Abad-Zapatero, C. (2018). *Acta Cryst.* D**74**, 321–331. 10.1107/S2059798318002838 PMC5892879 29652259

[bb20] Ziveri, J., Barel, M. & Charbit, A. (2017). *Front. Cell. Infect. Microbiol.* **7**, 96. 10.3389/fcimb.2017.00096 PMC5368251 28401066

