# Supplementary material for: Structural and biochemical characterization of the class II fructose-1,6-bisphosphatase from Francisella tularensis
Source: Acta Crystallogr F Struct Biol Commun. 2020 Oct 23;76(Pt 11):524–35. doi: 10.1107/S2053230X20013370 (PMC7605111; doi:10.1107/S2053230X20013370)
Supplement: Supplementary file 1 [file f-76-00524-sup1.pdf]

STRUCTURAL BIOLOGY COMMUNICATIONS

Volume 76 (2020)

Supporting information for article:

Structural and biochemical characterization of the class II fructose-1,6-bisphosphatase from *Francisella tularensis*

Anna I. Selezneva, Hiten J. Gutka, Nina M. Wolf, Fnu Qurratulain, Farahnaz Movahedzadeh and Celerino Abad-Zapatero

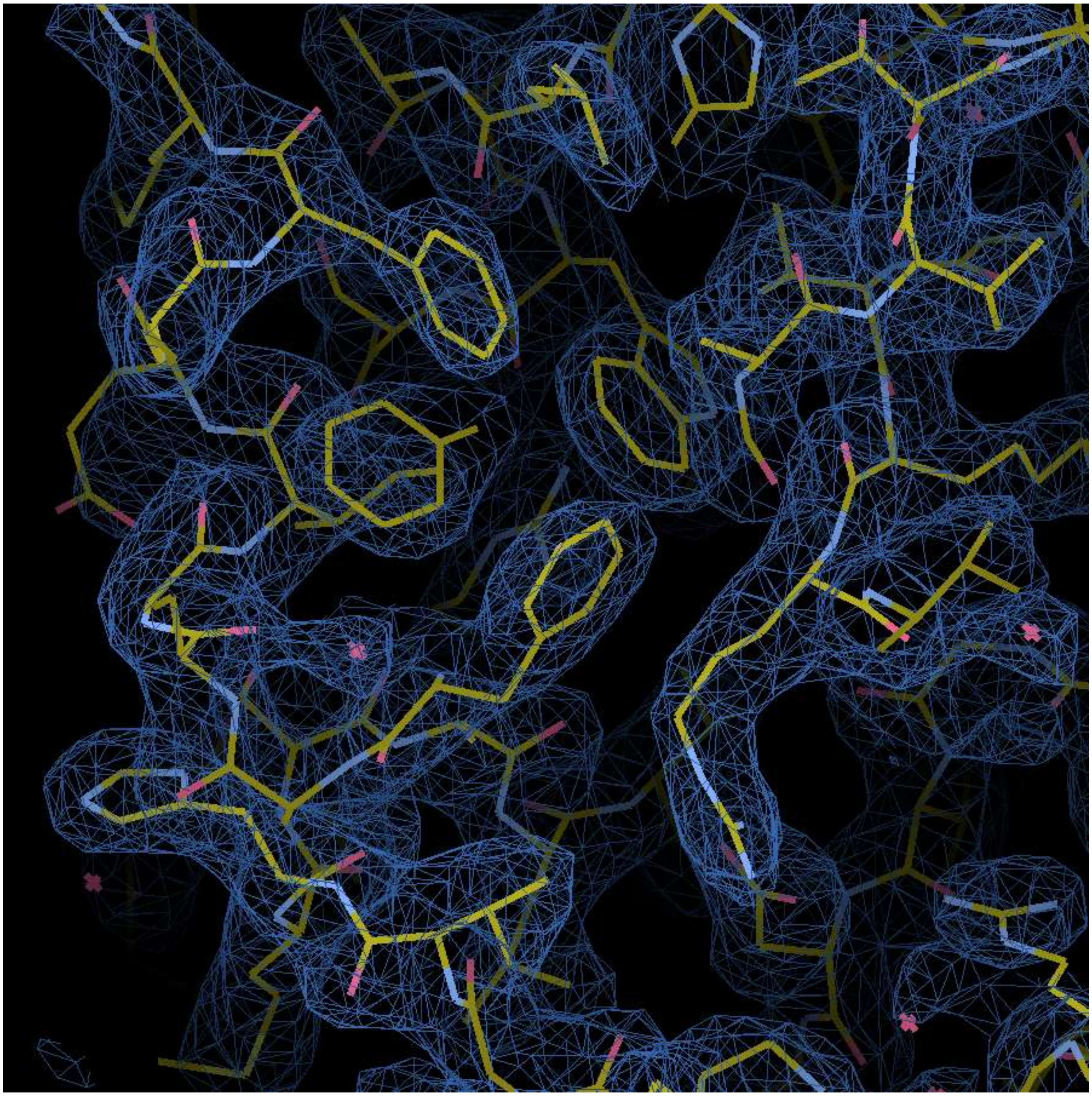

Figure S1 Sample of the electron density map in the final stages of refinement. Electron density showing the fitted polypeptide chain corresponding to the last eleven residues of the C-terminus forming a hydrophobic 'core' of *Ft*FBPaseII in Chain A. They fold around a very compact hydrophobic cluster that includes three Phe residues and is next to Trp21 in the N-terminal part of the chain including a short helical segment (H14) (see below). This stretch of eleven a.a. is unique to the sequence of related FBPases in Fig.3 and is conserved in the genus *Francisella*

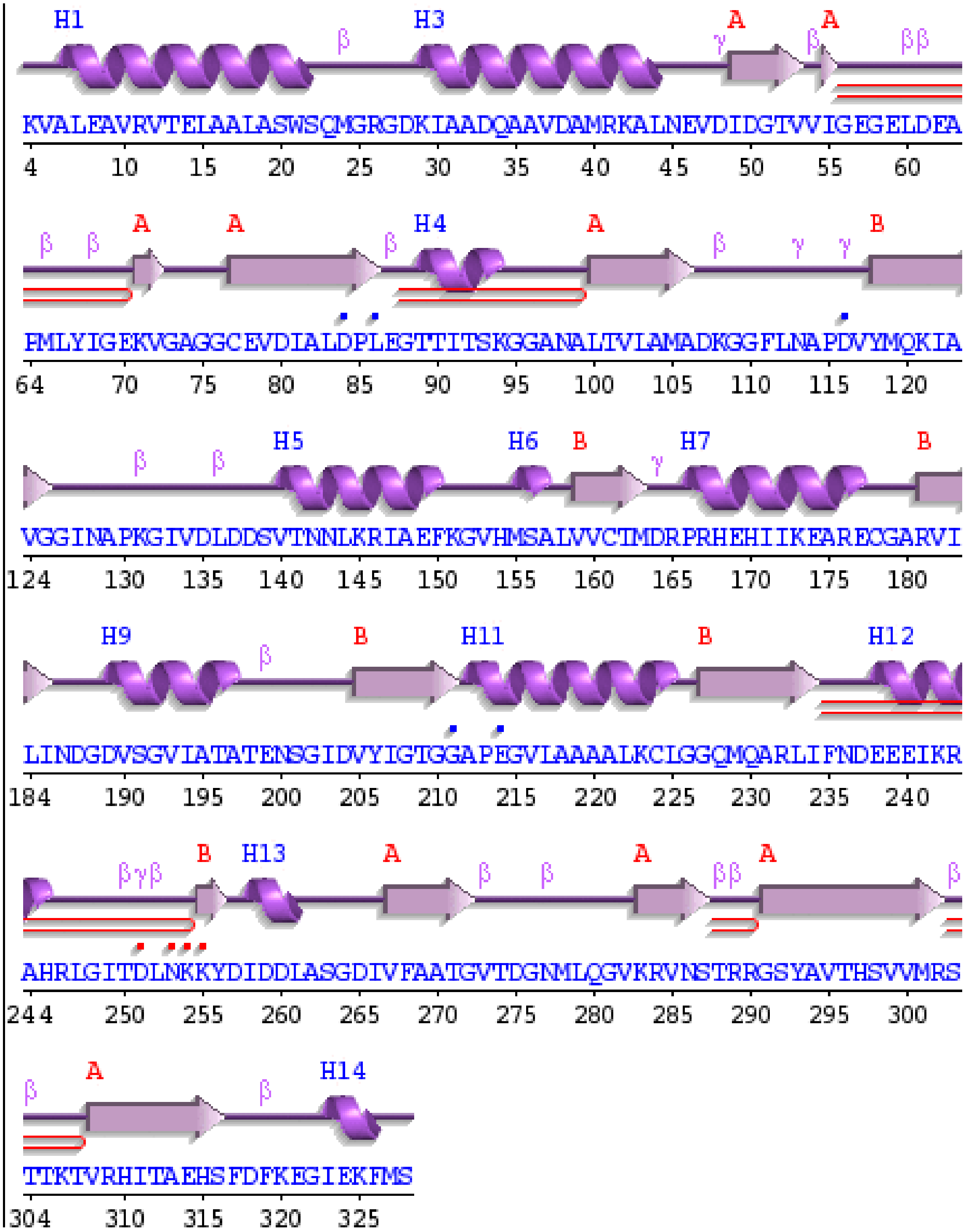

Figure S2 Secondary structure diagram of the Chain A of the *Ft*FBPaseII tetramer with the basic structural elements numbered along the polypeptide chain. The structure consists of two beta sheets (A, B). The central sheet (B) contains six strands surrounded on each side by four strands of the A sheet. The final helical segment (H14) is unique to *Ft*FBPaseII and is part of the C-terminal core, whose function is unknown. Figure prepared within PDBsum (Laskowski et al., 2017).

Table S1 Macromolecule-production information.

Source organism	*F. tularensis*
DNA source	*F. tularensis glpX* gene (FTF1631c)
Cloning strategy	Synthesized and cloned by CelTek Inc.
T89A forward primer	CCCGCTGGAAGGTGCGACCATTACCAGC
T89S forward primer	CCCGCTGGAAGGTTCGACCATTACCAGC
Expression vector	pET15b
Expression host	*E. coli* strain BL21(DE3)
Complete amino-acid sequence of the wild type construct produced	MGSSHHHHHHSSGLVPRGSHMNRKVALEAVR VTELAALASWSQMGRGDKIAADQAAVDAMRK ALNEVDIDGTVVIGEGELDEAPMLYIGEKVGAG GCEVDIALDPLEGTTITSKGGANALTVLAMADK GGFLNAPDVYMQKIAVGGINAPKGIVDLDDSV TNNLKRIAEFKGVHMSALVVCTMDRPRHEHIIK EARECGARVILINDGDVSGVIATATENSGIDVYI GTGGAPEGVLAAAALKCLGGQMQARLIFNDEE EIKRAHRLGITDLNKKYDIDDLASGDIVFAATG VTDGNMLQGVKRVNSTRRGSYAVTHSVVMRS TTKTVRHITAEHSFDFKEGIEKFMS

Table S2 Crystallization conditions for Crystal B.

Method	Sitting-drop vapor diffusion
Plate type	96-well plate
Temperature (°K)	291
Protein concentration (mg ml^{-1})	10
Buffer composition of protein solution	20 mM Tricine pH 7.8, 50 mM KCl, 1 mM $MgCl_2$, 0.1 mM DTT, 15% glycerol
Composition of reservoir solution	0.2 M sodium formate, 20% PEG 3350
Volume and ratio of drop	2n (1:1)
Volume of reservoir (ml)	100

Table S3 . Comparisons among the four different subunits of the FtFBPase

(a) Summary of RMSD[a] and average[b] distances (Å, upper diagonal) and B factor differences (RMSD[a]/averages[b]) ($Å^2$, lower diagonal) among the different chains (A-D) of the *Ft*FBPaseII tetramer.

	A	B	C	D
A	-	0.56<br>0.42	0.74<br>0.55	1.08[a]<br>0.82[b]
B	13.4[a]<br>7.9[b]	-	0.76<br>0.57	1.04<br>0.77
C	38.6<br>33.7	32.3<br>25.8	-	1.07<br>0.81
D	27.4<br>18.4	21.5<br>10.5	22.6<br>15.5	-

(b). Rotation angles among the different subunits in the *Ft*FBPaseII tetramer and *Mt*FBPaseII dimer in the asymmetric unit of the corresponding crystals. *Values in the three different columns for *Mt*FBPaseII correspond to the rotation angle transformations between the two chains (A>B) found in the a.u. for apo (6ayy) and the T84S-F6P (6ayu), T84A-F6P (6ayv) crystallographic complexes, respectively.

	A>B	A>C	A>D	B>C	B>D	C>D
*Ft*FBPase (°)	179.5	178.4	177.6	179.1	178.9	179.8
*Mt*FBPase*(°)	176.4	176.5	176.3			

Table S4 Summary of the kinetic parameters for class II FBPases related to this work.

Top: Kinetic parameters for the FtFBPaseII enzyme (wt and T89S mutant; T89A mutant was inactive) presented in this work in relation to the ones reported before. Bottom: corresponding values for the highly homologous MtFBPaseII present in *M. tuberculosis*.

**FtFBPaseII**	**K_m (µM)**	**V_{max} (µmol min^{-1} mg^{-1})**	**k_{cat} (s^{-1})**	**k_{cat}/K_m (s^{-1} mM^{-1})**	**Reference**
WT	11 ± 2	2.1 ± 0.1	1.2 ± 0.06	115	a
WT	17 ± 4	0.9 ± 0.1	0.55 ± 0.04	33	This work
T89S	4 ± 2.1	0.2± 0.03	0.12 ± 0.02	31	This work
**MtFBPaseII**					
WT	3.7 ± 0.1	3.5 ± 2.4	2.1 ± 1.5	570	b
T84S	5.7 ± 1.2	0.37 ± 0.26	0.22 ± 0.16	39	b

References: [a](Gutka et al .2017), [b](Bondoc et al. 2017)

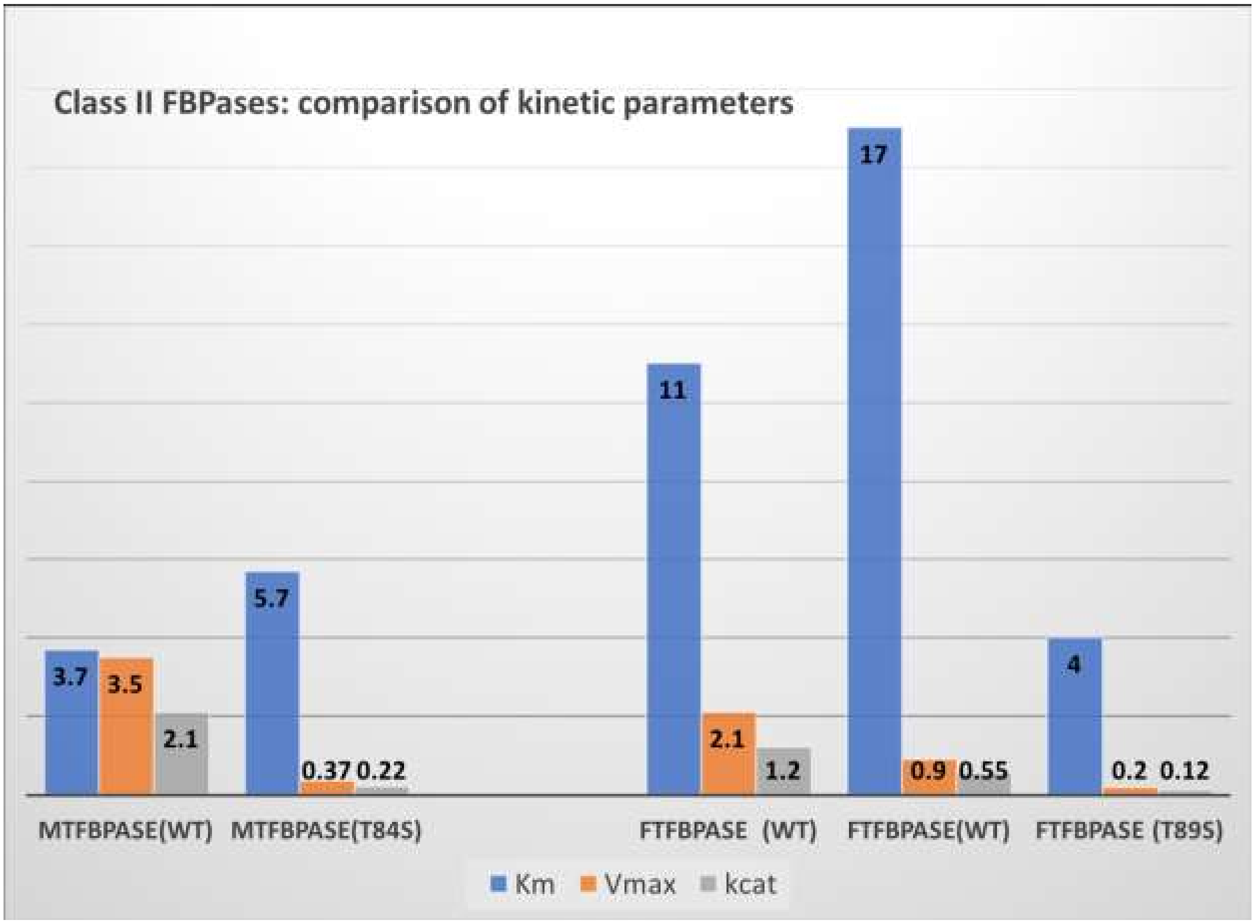

Figure S3 Graphical summary of the kinetic parameters listed in Table SI2 for the two class II FBPases discussed in this work: *Mt*FBPase and *Ft*FBPase (wild type and active site mutants) T84S and T89S, respectively homologous in the a.a. sequence.

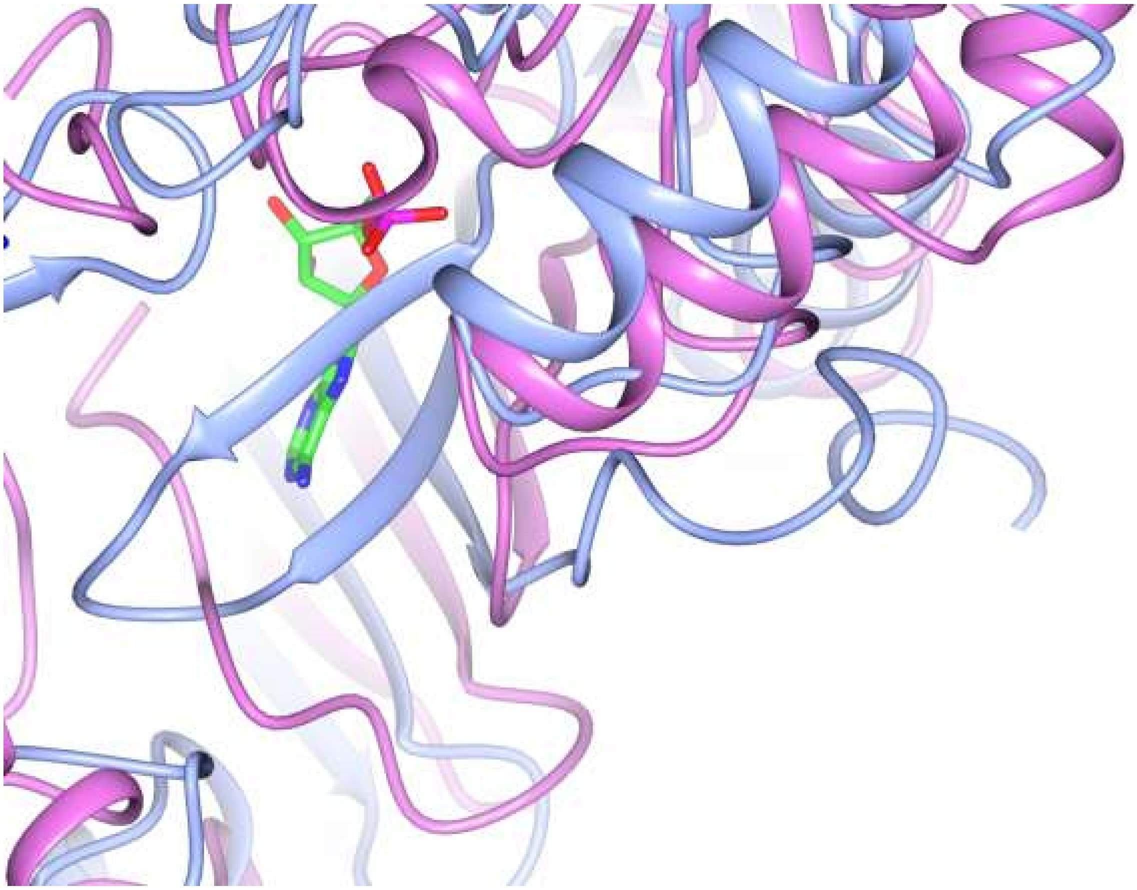

Figure S4 Differences between *Synechocystics* and *Ft*FBPaseII near the allosteric effector pocket. Comparison of the C-terminal extensions in 3rpl (purple) with the bound allosteric effector AMP (green-red-blue:carbon-oxygen-nitrogen) in the interior of the tetramer, in relation to the external extension of the same part of the polypeptide chain in *Ft*FBpaseII (blue). See above: C-terminus hydrophobic 'core' (Fig. SI1). The image also contrasts the shorter terminal β-strands in 3rpl with the longer corresponding two strands and hairpin-loop extension (front) in *Ft*FBPaseII (blue) that are 'curved' inwards towards the AMP pocket.
